# Engineered Bacteria-Nano Hybrid System: The Intelligent Drug Factory for Next-Generation Cancer Immunotherapy

**DOI:** 10.3390/pharmaceutics17101349

**Published:** 2025-10-20

**Authors:** Guisha Zi, Wei Zhou, Ling Zhou, Lingling Wang, Pengdou Zheng, Shuang Wei

**Affiliations:** 1 Department of Respiratory and Critical Care Medicine, National Health Commission (NHC) Key Laboratory of Respiratory Disease, Tongji Hospital, Tongji Medical College, Huazhong University of Science and Technology, Wuhan 430030, China; 2 Department of Pulmonary Critical Care Medicine, Wuhan Pulmonary Hospital (Wuhan Tuberculosis Prevention and Control Institute), Wuhan 430030, China; 3 Sino-German Joint Oncological Research Laboratory, Shanxi Bethune Hospital, Shanxi Academy of Medical Sciences, Taiyuan 030032, China; 4 Department of Respiratory and Critical Care Medicine, Shanxi Bethune Hospital, Shanxi Academy of Medical Sciences, Tongji Shanxi Hospital, Third Hospital of Shanxi Medical University, Taiyuan 030032, China

**Keywords:** engineered bacteria-nano hybrid system, tumor targeting, immune reprogramming, synthetic biology, controlled release

## Abstract

As one of the primary fatal diseases globally, cancer represents a severe threat to human health because of its high incidence and fatality rates. While traditional treatments including surgery, radiation, and conventional pharmacotherapy demonstrate therapeutic effects, they commonly suffer from issues like severe side effects, high rates of relapse, and immunosuppression. The advent of immune checkpoint inhibitors and targeted drugs has undoubtedly revolutionized cancer management and improved survival; however, a significant proportion of patients still encounter obstacles such as acquired resistance, an immunosuppressive tumor microenvironment, and poor drug delivery to avascular tumor regions. Recent integration of engineered bacteria with nanomaterials has offered novel strategies for cancer immunotherapy. Engineered bacteria feature natural tumor tropism, immune-stimulating properties, and programmability, while nanomaterials are characterized by high drug payload, tunable release profiles, and versatile functionality. This article reviews the application of hybrid systems integrating engineered bacteria and nanomaterials in cancer immunotherapy, exploring their potential for drug delivery, immunomodulation, targeted treatment, and smart responsiveness. The construction of an “intelligent drug factory” through the merger of bacterial biological traits and sophisticated nanomaterial functions enables precise manipulation of the tumor microenvironment and potent immune activation, thereby establishing a novel paradigm for the precise treatment of solid tumors. However, its clinical translation faces challenges such as long-term biosafety, genetic stability, and precise spatiotemporal control. Synergistic integration with therapies such as radiotherapy, chemotherapy, and immunotherapy represents a promising direction worthy of exploration.

## 1. Introduction

Ranking as the second leading cause of death worldwide, cancer imposes an increasingly severe epidemiological burden. Recent data from the International Agency for Research on Cancer (IARC) indicate approximately 20 million incident cancer cases and 9.7 million deaths worldwide in 2022, alongside a steadily rising incidence rate [1]. Conventional therapeutic paradigms face significant challenges due to the heterogeneity, predisposition to metastasis, and development of drug resistance inherent in solid tumors. Surgical resection, while potentially curative for localized lesions, is ineffective against occult micrometastases. Despite their extensive clinical application, radiotherapy and chemotherapy induce systemic adverse effects like myelosuppression and organ toxicity due to poor targeting specificity, and their efficacy is limited by inadequate penetration into tumor tissue and the development of resistance, making complete cancer cell clearance difficult. To break through the bottlenecks of conventional therapies, emerging treatment strategies are continuously emerging. Immune checkpoint inhibitors (ICIs) (such as PD-1/PD-L1 antibodies) represent a breakthrough by potentiating T cell-mediated anti-tumor immunity, yet their efficacy is confined to roughly 20–30% of patients [2]. While chimeric antigen receptor T cell (CAR-T) therapy demonstrates significant success in blood cancers, its application is constrained by the barrier posed by the solid tumor immune microenvironment [3]. Photodynamic therapy (PDT) and photothermal therapy (PTT), which depend on the spatiotemporal coordination of externally administered photosensitizers (PS) and laser light, have their therapeutic potential limited by poor tissue penetration depth [4,5]. In this context, bacteriotherapy has regained research interest owing to its distinctive tumor-homing ability—an approach dating back to the seminal 19th-century observation by William B. Coley that injections of inactivated streptococci could treat patients with inoperable cancers, unveiling the capacity of bacteria to elicit anti-tumor immune responses [6].

Contemporary studies reveal that the principal strength of bacteriotherapy originates from its innate affinity for the tumor microenvironment (TME). The characteristics of the TME—hypoxia, immunosuppression, and abundant necrotic core—furnish an ideal niche for colonization by facultative or obligate anaerobic bacteria (such as Escherichia coli, attenuated Salmonella, Listeria, Clostridium, and Lactobacillus) [7,8]. Bacteria accumulate intratumorally through passive entrapment (enhanced permeability and retention effect, EPR effect) and active chemotaxis (e.g., in response to tumor metabolites), subsequently exerting therapeutic effects through multiple mechanisms: directly lysing tumor cells, secreting toxins or enzymes to disrupt tumor vasculature, and crucially, their pathogen-associated molecular patterns (PAMPs) can activate Toll-like receptors (TLRs), alleviating the immunosuppressive state of the TME, promoting dendritic cells (DCs) maturation, cytotoxic T cell infiltration, and M1 macrophage polarization, thereby generating a systemic anti-tumor immune response [9,10]. However, natural bacterial therapy has significant limitations: highly virulent strains may cause sepsis, while excessive attenuation compromises efficacy; unstable gene expression leads to uncontrolled release of therapeutic molecules; the potential spread of antibiotic resistance genes poses ecological risks [11,12]. These safety and controllability deficiencies urgently need to be addressed through interdisciplinary technologies. While engineered bacteria demonstrate enhanced performance through reduced pathogenicity and inserted functional genes, their therapeutic payload remains limited, and the risk of virulence reactivation persists [9].

In contrast to conventional small-molecule cytotoxic agents, nanomaterials exhibit unique properties such as the quantum confinement effect, nanoscale effect, and surface-interface effect, endowing them with significant potential and applicability as drug carriers, imaging contrast agents, and gene delivery vectors [13,14,15]. Primarily, the distinctive EPR effect inherent to nanomaterials facilitates their selective accumulation within tumors, markedly enhancing the local concentration of therapeutic agents. Furthermore, their high surface-area-to-volume ratio allows for efficient loading of chemotherapeutic drugs or genetic therapeutics, and the encapsulated architecture shields the payload from enzymatic degradation [16,17]. Surface engineering strategies, such as conjugating the RGD peptide (Arg-Gly-Asp) or applying a PEG (polyethylene glycol) coating, can significantly enhance the pharmacokinetics and targeting specificity of nanocarriers. The RGD peptide mediates active targeting by binding to integrins overexpressed on tumors, while the PEG coating provides stealth properties to minimize clearance by the reticuloendothelial system and extend plasma half-life [18,19]. Notably, nanomaterials surpass the function of simple delivery vehicles; their innate acoustic, optical, electromagnetic, and thermal properties can mediate direct cancer cell killing or act synergistically with loaded drugs (e.g., immunogenic cell death (ICD) induced by gold nanoparticle-based PTT) [20]. However, nanocarriers also face challenges such as insufficient targeting efficiency, potential off-target toxicity, and poor biodegradability. The convergent fusion of synthetic biology and material science has yielded engineered bacteria-nano hybrid systems, which represent a category of programmable, intelligent drug factories operating in vivo, offering a comprehensive strategy for the precision advancement of bacteriotherapy. The hybrid system strategically addresses the limitations of individual engineered bacterial or nanotherapeutic approaches: microbial tropism directs nanomaterials to poorly vascularized tumor regions beyond the reach of passive delivery, and nano-encapsulation substantially improves drug loading capacity while permitting spatiotemporally controlled release through external stimulation, achieving enhanced localized drug accumulation with reduced off-target effects. Being a cutting-edge field focused on reprogramming biological systems using engineering concepts, synthetic biology enables exact control over the spatiotemporal behavior of engineered bacteria via the rational design of genetic components, modular reconstruction of metabolic circuits, and targeted refinement of genetic networks [21]. This technology exhibits multifaceted utility in oncology: engineered bacterial platforms utilizing Escherichia coli, attenuated Salmonella, Bifidobacterium, and Streptococcusare endowed with enhanced therapeutic capabilities beyond their native counterparts through the implementation of intelligent genetic circuits [22,23,24]. These engineered bacteria can precisely execute therapeutic commands within the TME: after selective colonization, they express cytokines (e.g., TNF-α, IL-12), cytotoxic peptides, and prodrug-converting enzymes (e.g., cytosine deaminase) in situ, while also responding to endogenous signals like low pH and hypoxia or exogenous stimuli like light and heat to achieve on-demand drug release [25,26,27,28]. Concurrently, material science contributes by employing functional nanomaterials (such as gold nanorods and pH-responsive hydrogels) to confer upon bacteria the ability for environmentally triggered drug delivery. This “living-inorganic” hybrid system not only fortifies the robustness of engineered bacteria for crossing physiological barriers but also substantially augments tumor eradication efficacy via signal amplification cascades and multimodal response mechanisms, heralding the advent of an intelligent, integrated, and precise new epoch for bacteriotherapy.

However, despite its considerable potential in cancer therapy, the clinical translation of engineered bacteria-nano hybrid systems faces significant challenges. Long-term biosafety remains a primary concern, as the exogenous “living” components in vivo may trigger uncontrolled immune responses or potential toxicity. Concurrently, engineered genetic circuits may undergo mutation or loss during bacterial proliferation, leading to diminished therapeutic efficacy or unpredictable behaviors. Furthermore, achieving precise spatiotemporal control over therapeutic activation while minimizing off-target effects remains a formidable obstacle. Synergistic integration with established therapies such as radiotherapy, chemotherapy, and immunotherapy represents a promising direction worthy of exploration.

This review summarizes the applications of engineered bacteria-nano hybrid systems in tumor immunotherapy, with a particular emphasis on novel strategies for tumor-targeted delivery, immunomodulation, controlled drug release, and biocompatibility enhancement, alongside therapeutic approaches that achieve spatiotemporal integration with chemotherapy, radiation, immunotherapy, PDT, and PTT.

## 2. Mechanisms of Engineered Bacteria in Cancer Treatment

### 2.1. Tumor Targeting

A principal strength of engineered bacteriotherapy is its innate tumor-homing ability, which forms the essential feature differentiating it from conventional pharmaceutical agents. Preclinical research validates that bacteria administered via intravenous injection exhibit specific accumulation within tumor tissue, achieving concentrations several hundred to thousand-fold higher than those found in healthy organs [29]. In murine xenograft models (e.g., of breast cancer, melanoma, pancreatic carcinoma) and orthotopic tumor models, administration of strains including attenuated *Salmonella* and *Escherichia coli* via tail vein injection resulted in preferential bacterial colonization at the tumor site, as detected by bioluminescent imaging and colony-forming unit counts, concomitant with a marked reduction in bacterial burden in organs such as the liver and spleen over time [30,31]. This phenomenon of selective accumulation unveils the distinctive interactive mechanisms operating between bacteria and the TME.

The tumor-targeting proficiency of engineered bacteria originates from the graded coordination and dynamic integration of multi-tiered biological mechanisms. Passive entrapment serves as the initial step for overcoming physical barriers, where pathologically enlarged inter-endothelial cell junctions and a disorganized basement membrane create a sieve-like structure, leading to bacterial retention inside tumors through the EPR effect [32,33]. Subsequently, active penetration mechanisms enhance targeting accuracy; elevated levels of TNF-α within the TME promote bacterial adhesion via induction of endothelial adhesion molecule expression, and stimulate matrix metalloproteinases to breakdown the extracellular matrix (ECM), thereby facilitating deep migration of bacteria into the tumor stroma [34]. Bacterial adaptation to the biochemical signature of the TME is crucial for ensuring targeting specificity. The hypoxic core attracts facultative anaerobes by activating hypoxia-responsive systems, guiding their directed migration [35]; the mildly acidic milieu induces acid resistance pathways to preserve cellular homeostasis, and tumor cell-derived metabolites like lactate and glutamine activate amino acid transporters, fueling bacterial exponential growth [36,37]. This adaptation demonstrates stringent spatial specificity; for example, spores of Clostridium difficile germinate exclusively in the hypoxic tumor core while maintaining dormancy in non-malignant hypoxic tissues, thereby molecularly circumventing off-target effects [38]. Moreover, as self-propelling living entities, bacteria utilize flagellar motors to power rapid movement, and their chemoreceptors precisely detect tumor-derived chemical gradients, achieving profound infiltration into the dense tumor stroma [39,40]. This process acts in concert with the immune-evasive nature of the TME: myeloid-derived suppressor cells (MDSCs) deplete microenvironmental arginine via high arginase expression, and regulatory T cells (Tregs) secrete immunosuppressive cytokines that impair macrophage phagocytosis, together fostering an immune-sanctuary niche that markedly extends the persistence of the bacterial burden within the tumor [41].

The subsequent proliferation of viable bacteria following successful colonization is pivotal for amplifying the anti-tumor efficacy. The capacity of engineered bacteria for self-replication inside tumors can dramatically elevate the local concentration of therapeutic agents, establishing a persistent in situ bioproduction platform [42]. For instance, attenuated *Salmonella* A1 strain proliferates at a rate four orders of magnitude greater in tumor tissue compared to normal tissue; by day 15 post-injection, the tumor-to-liver bacterial ratio was as high as 10,000:1, bacteria were eradicated from healthy tissues, yet the intratumoral bacterial population persisted at levels sufficient for therapeutic activity [43]. This trait of selective replication not only enhances the efficiency of local drug production but also furnishes the biological rationale for developing single-dose, sustained-release therapeutic regimens.

### 2.2. Tumor Killing

#### 2.2.1. Direct Killing

Engineered bacteria mediate potent tumor eradication via multifaceted mechanisms, with their direct cytotoxicity routes exhibiting precise cell-disrupting abilities. After invading tumor cells, bacteria replicate exponentially inside the cytosol, leading to physical distension and rupture of the host cellular membrane [44]. Concurrently, bacteria secrete membrane-active toxins like α-hemolysin and phospholipase C that hydrolyze membrane phospholipids and create transmembrane channels, compromising membrane integrity, resulting in ionic dyshomeostasis and efflux of cellular contents, thereby inducing oncotic death of tumor cells [44,45]. The generation of a reactive oxygen species (ROS) storm represents a crucial cytotoxic modality. For example, *Listeria* species activate NADPH oxidase to generate superoxide anions (O_2_^−^•) and hydrogen peroxide (H_2_O_2_), provoking endoplasmic reticulum stress and mitochondrial impairment. This not only mediates direct tumor cell killing but also elicits DCs-driven adaptive immune responses through the induction of ICD markers like surface-exposed calreticulin [46]. Beyond damaging cancer cells, the ROS storm facilitates the maturation of antigen-presenting cells (APCs) via the release of DAMPs like HMGB1 ensuing from oxidative DNA damage, thus converting a localized cytotoxic effect into a systemic immune reaction [39]. Additionally, bacteria directly trigger the Caspase cascade via secretion of pro-apoptotic factors, or indirectly provoke macrophages to release death ligands like TNF-α, thereby exerting dual control over the apoptotic signaling network. As an example, engineered *Escherichia coli* Nissle 1917 (EcN), which expresses Colicin E3 intratumorally, directly initiates the Caspase-3/7 cascade, enhancing CD8^+^ T cell infiltration and antigen cross-presentation [47]. ATP released during the apoptotic process serves as a chemoattractant to recruit immune cells, and the tumor antigens harbored within apoptotic bodies augment cross-presentation efficacy, establishing a reinforcing immune cycle. This immunomodulatory reprogramming advances bacteriotherapy from mere direct cytotoxicity towards holistic modulation of the tumor microenvironmental ecology.

#### 2.2.2. Targeting the Tumor Vascular System

The growth and metastasis of tumors are critically reliant on the establishment and sustenance of their vascular network. Consequently, targeting the tumor vasculature has emerged as a pivotal strategy in anticancer treatment. Engineered bacteria demonstrate distinctive promise in this arena. Engineered bacteria can potently suppress tumor blood vessel formation and dilation through the expression of vascular endothelial growth factor (VEGF) antibodies or antagonists of its receptors. For example, genetically modified *Bifidobacterium longum* secreting the antiangiogenic factor tumstatin markedly inhibited endothelial cell proliferation and triggered tumor cell apoptosis in CT26 murine tumor models [48]. Simultaneously, via surface decoration with targeting moieties like antibodies, ligands, or nanoparticles, engineered bacteria gain the ability to specifically recognize and adhere to receptors expressed on tumor vascular endothelial cells, enabling their precise homing to the tumor site [49]. This approach not only augments the accumulation of bacteria within tumors but also minimizes potential off-target effects on healthy tissues. In addition, engineered bacteria can secrete cytokines such as IFN-γ and TNF-α, which activate signal transduction pathways in tumor endothelial cells, leading to their apoptosis and enhanced vascular permeability, thus facilitating improved infiltration of immune cells and therapeutic agents into the tumor mass [26]. This mechanism results in the collapse of the tumor microcirculation, inducing nutrient starvation and accumulation of metabolic waste, working in concert with direct cytotoxicity to accomplish complete elimination of solid tumors.

### 2.3. Production of Therapeutic Molecules

The natural biological characteristics of bacteria allow for the sustained production and release of therapeutic agents within tumors, overcoming the delivery limitations inherent to conventional chemotherapeutic drugs. In contrast to the systemic delivery paradigm of conventional chemotherapy, in situ synthesis mediated by engineered bacteria provides two key benefits: its targeting specificity stems from the intrinsic ability of bacteria to specifically colonize the TME, guaranteeing high local concentrations of therapeutics at the tumor site, while substantially minimizing toxicity to healthy tissues, thereby comprehensively tackling the clinical issues of poor delivery efficiency and a narrow therapeutic index associated with traditional chemotherapy [50]. Engineered bacteria are capable of synthesizing a diverse array of therapeutic agents, including bacterially secreted toxins, recombinant effector proteins derived from genetic engineering, nucleic acids that modulate gene expression, and metabolizing enzymes that catalyze prodrug activation [51].

The mechanism underpinning the enhanced efficacy of in situ production depends on a profound integration of bacterial biological processes with the TME. Bacteria achieve specific accumulation within tumors via passive entrapment and active chemotaxis, resulting in local drug concentrations substantially exceeding systemic circulatory levels, thereby surmounting the limitation of poor penetration depth characteristic of conventional nanocarriers [52]. Even more critical is the sustained replicative capacity of viable bacteria intratumorally; engineered bacteria undergo division approximately every 20–30 min within the TME, and the exponentially amplifying bacterial community persistently manufactures therapeutic molecules, leading to a time-dependent, dynamic escalation of the local drug concentration [50]. For instance, upon intratumoral colonization, EcN sustains proliferation and releases nanobodies, enabling an exponential increase in the local concentration of the therapeutic agent [28]. Following a single administration, the persistent expansion of bacteria inside the tumor markedly suppresses tumor growth; this self-reinforcing mode of drug production facilitates sustained therapy from a single dose, dramatically improving antitumor potency.

Technological innovations in synthetic biology further endow in situ synthesis with intelligent responsive characteristics. Through the incorporation of environment-responsive promoters—for instance, the acidic pH-activated STM1787 promoter for specific gene expression in the acidic TME, the hypoxia-inducible PfnrS promoter for targeted activation in hypoxic niches, and the optically controlled pDawn promoter for exogenous light-mediated regulation of transcription—engineered bacteria gain the ability to initiate the synthesis of therapeutic agents in a demand-driven manner [53,54]. Facultative anaerobic bacteria like *Escherichia coli* and *Salmonella Typhi* can be engineered into hypoxia-responsive bacterial vectors [55]. Xu et al. employed codon optimization for a tumor-associated antigen sequence and exploited the *Salmonella* Type III Secretion System to directly transport a recombinant neuroactive peptide into the cytosol of APCs, observing complete tumor regression in a mouse model [56]. This precise spatiotemporal regulation capability confines drug synthesis strictly to the tumor site, minimizing off-target toxicity to the greatest extent, while achieving efficacy maximization through local high-concentration drug exposure.

### 2.4. Immune Modulation and Activation

In engineered bacteria-mediated cancer immunotherapy, immunogenicity represents a critical attribute, denoting the capacity of the bacteria or their constituents to potently activate the host immune system. Engineered bacteria elicit systemic antitumor immune responses via surface-exposed PAMPs. As exogenous immune activators, bacterial components lipopolysaccharide (LPS) and flagellin B (FlaB) reverse immunosuppression via the TLR4 and TLR5 signaling pathways, respectively [57]. FlaB activates NK cells to promote perforin-dependent tumor cell killing, while simultaneously triggering M1 macrophage polarization, enhancing the secretion of nitric oxide and pro-inflammatory factors such as IL-12 and TNF-α, directly inhibiting tumor growth [34]. LPS, on the other hand, activates macrophages via the TLR4 pathway, inducing an inflammatory cytokine storm and reshaping a pro-inflammatory microenvironment. This dual activation strategy efficiently converts immune-excluded tumors into those that are infiltrated by immune cells. Furthermore, engineered bacteria can also directly disable the function of immunosuppressive cells. For example, *Listeria* weakens the immunosuppressive activity of Tregs by downregulating their IL-10 expression, while also reducing the arginase expression of MDSCs, reversing the immunosuppressive state of the microenvironment [58]. This immune remodeling, synergizing with the activation by PAMPs, achieves increased CD8^+^ T cell infiltration in refractory tumor models such as pancreatic cancer and melanoma, offering a novel strategy to overcome immunotherapy resistance in solid tumors [28,30]. Furthermore, engineered bacteria can also modulate the expression of MHC molecules on the surface of cancer cells, improving the efficiency of antigen presentation and consequently enabling more effective recognition and attack of tumor cells by T cells. This regulatory effect not only enhances the primary immune response but also provides a foundation for the formation of immune memory [59].

### 2.5. Controlled Release and Spatiotemporal Precision

Quorum Sensing (QS) is an internal communication mechanism within bacterial populations through which bacteria can sense their own population density and accordingly coordinate group behavior to achieve population effects [60]. In engineered bacteria-mediated tumor immunotherapy, QS serves as a fundamental mechanism enabling precise spatiotemporal regulation of therapeutic agents. This system operates based on bacterial secretion of autoinducers like acyl-homoserine lactone (AHL), whose concentration gradients build up concomitantly with microbial proliferation. Upon reaching a critical density threshold, autoinducers bind to specific sequences, initiating downstream transcriptional cascades that facilitate programmed control of bacterial activities [61]. This property holds particular promise for oncology—as the TME represents the sole anatomical site where bacteria attain sufficiently high densities in vivo, the QS system can restrict the production and release of therapeutics exclusively to the tumor locale [62]. QS-mediated precision control is implemented via two principal engineering paradigms: a tumor-specific recombinant protein expression mode, wherein therapeutic genes are placed under the control of QS-inducible promoters to achieve tightly constrained local production within tumors [63]; and a bacterial density-threshold triggering mode, which employs a predefined microbial density threshold as an activation switch for therapy, ensuring dynamic coupling between drug release and the intrinsic bacterial biomass within the tumor [64]. This dual spatiotemporal control mechanism strictly confines the therapeutic activity of bacteria to the interior of the tumor, avoiding systemic toxicity, while maintaining effective drug concentrations through continuous in situ administration. QS-mediated intelligent release possesses self-feedback amplification characteristics. During bacterial proliferation, autoinducers produced by LuxI accumulate, activating the LuxR transcription factor and establishing a positive feedback loop that escalates the rate of therapeutic molecule synthesis in parallel with population growth [65]. In metastatic tumor models, engineered *Salmonella* expressing QS-regulated apoptosis-inducing ligand TRAIL initiated the therapeutic program only when colonization in metastatic lesions reached a critical density, extending mouse survival without observed hepatotoxicity [65]. This self-driven and self-regulating drug delivery system signifies a paradigm transition for bacteriotherapy towards closed-loop intelligent control.

The Synchronized Lysis Circuit (SLC) is a genetic circuit constructed using synthetic biology techniques, which enables the synchronized release of drugs via precise temporal control of bacterial lysis, representing a core mechanism for pulsed drug administration by engineered bacteria [66]. Upon AHL concentration attaining a critical threshold, it induces the expression of lytic proteins, provoking synchronous bacteriolysis at the peak of proliferation, which instantaneously liberates intracellular therapeutics including cytotoxins and immunomodulators [64]. The residual bacterial cohort that survives lysis recommences AHL synthesis, establishing a dynamic “proliferation-accumulation-lysis-regeneration” cycle that facilitates periodic burst-like drug release [67]. The core advantage of SLC is its ability to overcome the spatiotemporal constraints of conventional drug delivery; a single injection facilitates multiple rounds of intratumoral pulsed dosing, elevating the local drug concentration within the tumor by over 10-fold within a brief period [68]. In a glioblastoma model, SLC-equipped engineered bacteria harboring the 5-fluorouracil prodrug-converting enzyme mediated a 75% reduction in tumor size via three pulsatile release events, markedly superior to the continuous delivery group (which exhibited only a 35% reduction) [28]. This self-perpetuating pulsatile drug delivery system offers a transformative solution for deeply situated and therapy-resistant tumors by preventing premature drug catabolism and sustaining concentrations within the therapeutic window.

Nevertheless, the clinical translation of engineered bacteriotherapy faces significant hurdles. Key challenges encompass safety concerns, including the risk of systemic infection or excessive inflammatory responses; genetic instability, which can compromise therapeutic efficacy or lead to unintended phenotypes; and off-target immune activation that may cause premature bacterial clearance or detrimental systemic inflammation. Concerted research is focused on addressing these issues through the development of more robust genetic circuits, improved immune-evasive designs, and rational combination therapies.

## 3. Synthetic Biology-Driven Engineered Bacteria-Nano Systems

**Figure 1:**
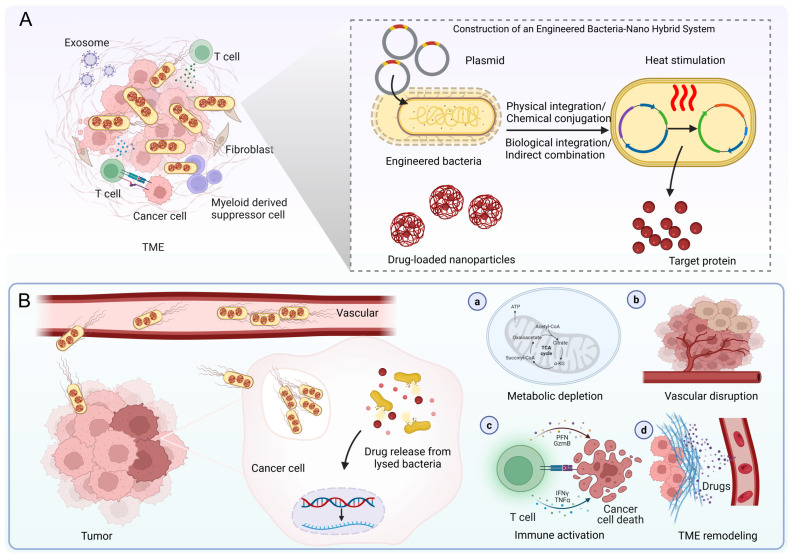
Schematic illustration of cancer therapy using engineered bacteria-nano hybrid system. (**A**) Construction of the hybrid system. (**B**) Mechanisms of cancer therapy, comprising metabolic depletion, vascular disruption, immune activation, and TME remodeling. TME, tumor microenvironment. Figure created with BioRender (https://www.biorender.com; accessed 10 September 2025).

Hybrid systems integrating engineered bacteria and nanomaterials, conceived through synthetic biology principles, represent a new class of anticancer platforms that have undergone a transformative evolution from passive delivery vehicles to “living drug factories”. The pivotal advance resides in the use of genetic circuit programming to confer upon bacteria smart responsiveness, precise tumor-homing properties, and multifaceted cooperative therapeutic actions [69]. Synthetic biology approaches markedly improve the targeting specificity, safety profile, and controllability of engineered bacteria via the rational design of genetic circuits. Multidimensional stimulus-responsive systems (e.g., pH-responsive, optically controlled, thermally inducible, and oral controlled-release) enable spatiotemporally precise release of therapeutic molecules (Figure 1). Concurrently, CRISPR-mediated virulence gene deletion and metabolic engineering enhance tumor colonization while minimizing systemic toxicity [70,71,72]. This “living drug factory” paradigm of bacteria-nano systems surmounts the penetration barriers inherent to conventional drug delivery. When integrated with synergistic strategies like catalytic therapy, oral vaccination, and nanocoating-mediated controlled release, it can address tumor heterogeneity and reprogram the immune microenvironment [73,74,75]. A summary of representative clinical studies utilizing engineered bacterial-nano hybrid systems for cancer therapy is provided in Table 1.

### 3.1. Construction Strategies for Engineered Bacteria-Nano Systems

Effective synergistic tumor therapy requires the co-aggregation of engineered bacteria and nanodrugs in the target area. Conventional nanocarriers suffer from limited targeting efficacy owing to elevated tumor interstitial pressure and impaired permeability, while bacteria exploit their hypoxia-directed chemotaxis, self-propulsion capability, and TME tropism to overcome biological barriers and infiltrate the tumor core. Consequently, the construction of bacteria-nanomaterial complexes emerges as a crucial strategy, enabling co-delivery via four principal methodologies: physical, chemical, biological, or indirect combinatorial approaches. These approaches must minimize detrimental effects on bacterial viability, ultimately provoking synergistic antitumor effects within the tumor (such as the coupling of PTT with immune activation).

Physical approaches employ non-covalent interactions, including electrostatic forces and modulated membrane permeability, to associate nanomaterials with the bacterial surface [91]. Representative techniques encompass: electroporation, which enhances membrane permeability via an applied electric field to facilitate drug internalization; electrostatic adsorption, utilizing charge-charge interactions for material association; and dip-coating or membrane coating, whereby materials are directly deposited onto the bacterial surface [91,92]. For instance, Ding et al. developed a hybrid system comprising black phosphorus quantum dots and engineered Escherichia coli designed to target hypoxic tumors and potentiate near-infrared (NIR) PDT. This system exhibited pronounced tumor inhibition and favorable biocompatibility in both in vitro and in vivo studies, furnishing proof-of-concept for the strategy of combining “bacterial carriers with photosensitizing nanomaterials” to counteract tumor hypoxia and improve PDT outcomes [93]. Although these methods are simple, rapid, and convenient to operate, electroporation may damage bacterial viability, and electrostatic binding is susceptible to ionic interference in vivo, resulting in insufficient stability. To improve conjugation stability, chemical strategies facilitate robust coupling between bacteria and nanomaterials via covalent linkages like amide bonds and Schiff base formations [94]. Classical methodologies encompass carbodiimide-mediated condensation and click chemistry, whereas in situ growth techniques (such as biomineralization of nanoparticles on bacterial surfaces) can simplify the procedure [95]. For example, after *Salmonella* YB1 was anchored with indocyanine green (ICG) nanoparticles via amide bonds, its tumor accumulation increased 14-fold, synergistically enhancing efficacy through PTT and immune activation [96]. Chen et al. reported a bio-hybrid platform (PTB@ZIF-90/MB) constructed by conjugating self-mineralizing photothermal bacteria with a mitochondria-targeting metal-organic framework (ZIF-90) loaded with methylene blue (MB) through acid-labile imine linkages. This platform integrates the innate tumor-targeting ability of bacteria with efficient NIR photothermal properties, markedly improving photothermal tumor ablation efficacy while exhibiting controllable in vivo biocompatibility [97]. Although this strategy enhances in vivo delivery reliability, chemical modification may lead to non-specific adsorption and increase preparation complexity.

In contrast to chemical modification, biological strategies optimally preserve bacterial viability by harnessing biospecific interactions or metabolic processes to accomplish high-affinity bioinspired assembly [98]. This methodology includes bioaffinity coupling, metabolic labeling, and genetic engineering (enabling engineered bacteria to autonomously produce therapeutic nanoparticles). For example, Suh constructed NanoBEADS by combining tumor-targeting attenuated *Salmonella* VNP20009 with poly (lactic-co-glycolic acid) (PLGA) nanoparticles through streptavidin-biotin coupling, increasing the nanocarrier load in solid tumors by approximately 100-fold, significantly improving efficacy and reducing systemic side effects [99]. Liu et al. covalently integrated the aggregation-induced emission PS MA into the cell wall of attenuated *Salmonella* VNP20009 through metabolic labeling, constructing a “living therapeutic agent” that can colonize tumor sites in vivo and release plasmids under light control. The results showed that under light irradiation, MA produced ROS that damaged the bacterial membrane, prompting the release of the carried VEGFR2 plasmid and its expression in host cells, inducing a T cell-mediated anti-angiogenic autoimmunity response and significantly inhibiting breast cancer tumor growth, while demonstrating good tumor targeting, controllability, and safety at the animal level [100]. To achieve efficient accumulation of nanodrugs at the tumor site, indirect combination strategies combine engineered bacteria with various large-scale carriers such as hydrogels and polyelectrolyte vesicles, providing an effective platform for nanodrug delivery [101,102]. Li et al. constructed a bio-inorganic hybrid microswimmer by encapsulating *Escherichia coli* with ZIF-8 metal-organic framework (MOF) nanoparticles. This system exhibited chemotaxis-guided targeted active drug delivery and augmented antitumor potency in vitro, alongside demonstrating potential for extended residence time within the murine bladder [103]. Moreover, magnetic bacteria can precisely deliver drug-loaded liposomes to the hypoxic regions of tumors (permeability up to 55%) under the guidance of an external magnetic field, while hydrogel co-delivery systems can simultaneously trigger drug release and immune system activation through the action of bacterial lipases [104]. This precise drug delivery and regulation mechanism provides a more effective and intelligent solution for tumor therapy.

### 3.2. Living Factories and Controlled Release

**Figure 2:**
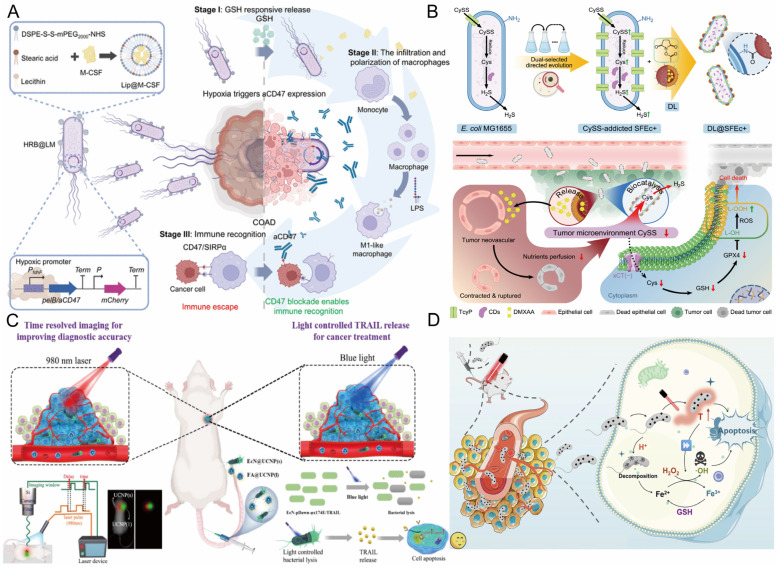
(**A**) Schematic illustration of the hypoxia-responsive HRB@LM system for cancer therapy via modulation of aCD47 expression. Reproduced with permission from Ref. [108]. Copyright 2025, The American Association for the Advancement of Science. (**B**) Schematic illustration of the construction of DL@SFEc+ and its mechanism in cancer therapy. Reproduced with permission from Ref. [109]. Copyright 2025, Elsevier. (**C**) Mechanism of blue laser light-induced lysis of engineered bacteria for controlled release of TRAIL, enabling smart drug delivery and cancer therapy. Reproduced with permission from Ref. [119]. Copyright 2022, American Chemical Society. (**D**) Mechanism of synergistic cancer therapy combining hypoxia-responsive FeS@SRB with PDT and PTT. Reproduced with permission from Ref. [9]. Copyright 2025, Springer Nature. PDT, photodynamic therapy; PTT, photothermal therapy; TRAIL, tumor necrosis factor-related apoptosis-inducing ligand.

In engineered bacteria-mediated tumor immunotherapy, the expression of therapeutic molecules is the core link in constructing a “living drug factory”. This approach utilizes modular plasmid design to integrate genes for specific therapeutic agents (such as tumor antigens or immune modulators) into engineered bacteria, enabling versatile programming of therapeutic functionalities [105]. Artificially designed plasmid systems integrate elements such as promoters, therapeutic genes, replicons, and selection markers, and are transfected into bacterial chassis (e.g., attenuated *Salmonella*, EcN) via electroporation or conjugation, forming stably inherited expression systems that enable engineered bacteria to continuously synthesize and release high concentrations of bioactive molecules at the tumor site [106]. Zhang et al. employed *Escherichia coli* as a bio-scaffold for in situ polymerization-induced self-assembly (iPISA), subsequently forming composite nanomaterials with gold (Au). They found that the bacteria absorb and enrich Au inorganic precursors, mediate the formation of embedded polymer nanoparticles, and consequently form polymer-gold composite materials in the biohybrid system. Moreover, leveraging these in situ generated composite nanomaterials, the microbial system can manufacture high-value chemicals through processes like photocatalysis [107]. Fan’s team constructed a hybrid system of nanoliposomes and engineered *Escherichia coli* (HRB@LM) (Figure 2A), wherein attenuated Escherichia coli was programmed for in situ synthesis and sustained release of anti-CD47 antibodies within the hypoxic TME, coupled with surface attachment of redox-responsive liposomes carrying M-CSF, enabling precise tumor targeting, macrophage recruitment, and M1 polarization (Table 2) [108]. Employing a dual-selection directed evolution approach, Wang et al. constructed an *Escherichia coli* strain, SFEc+, with enhanced capacity for uptake and degradation of L-cystine/cysteine (CySS), and chemically conjugated it to liposomes (DL) loaded with the antiangiogenic agent DMXAA, fabricating a living bacterium-nanomedicine biohybrid designated DL@SFEc+ (Figure 2B). After intravenous administration, DL@SFEc+ specifically targeted and colonized tumor tissue, inducing contraction and rupture of tumor neovasculature through in situ released DMXAA, thereby effectively blocking the tumor’s nutrient supply. Simultaneously, the enhanced CySS biocatalytic cascade reaction of SFEc+ continuously consumed the CySS reserves in the TME. This dual mechanism of action induced redox imbalance and lipid peroxidation in tumor cells, leading to significant tumor suppression in mouse tumor models (including spontaneous intestinal adenocarcinoma and refractory pancreatic cancer) with good biocompatibility [109]. Similarly, Fan et al. constructed a biohybrid microrobot based on the oral-intestinal commensal bacterium *Veillonella atypica* (VA) (VA-SAM@BTO), by attaching *Staphylococcus aureus* membrane-coated BaTiO3 (SAM@BTO) to the VA surface via copper-free click chemistry, achieving dual targeting and deep penetration against orthotopic colorectal cancer after oral administration, with an inhibition rate of approximately 90% in the orthotopic colorectal cancer model [110]. Compared to traditional systemic administration, this technology avoids multiple dosing while significantly increasing the local drug concentration in the tumor, systematically addressing the clinical challenges of low drug delivery efficiency and a narrow therapeutic window. As previously noted, the therapeutic agents produced by engineered bacteria include a diverse range of functional entities, including cytokines, cytotoxins, prodrug-converting enzymes, siRNA, and ICIs antibody fragments.

Promoter-mediated environmental responsiveness is pivotal for spatiotemporal control, with its essential function being the sensing of external cues and environmental alterations to initiate target molecule expression spatiotemporally, thus restricting drug production exclusively to the tumor site [117]. Acidic pH-inducible promoters activate transcription of therapeutic genes within the acidic TME (pH < 6.8), directly capitalizing on the lactate-rich nature of solid tumors [111]. Qin et al. employed an acid-inducible attenuated *Escherichia coli* strain to express cytolysin A (ClyA), establishing a tumor therapeutic platform. Expression of ClyA within the acidic milieu induces thrombus formation, severing the nutrient supply to cancer cells and resulting in membrane poration and cellular injury. The experimental results demonstrated that this approach successfully curtailed tumor proliferation by 79% and markedly inhibited tumor metastasis in xenograft murine models [118]. Zhang et al. constructed an optically controlled engineered bacteria system utilizing upconversion nanoparticles (UCNPs) for time-resolved imaging (TRI) (Figure 2C). They employed engineered EcN harboring a blue light-responsive genetic circuit that triggers lysis and release of TNF-related TRAIL upon blue light exposure, enabling targeted tumor cell killing [119]. Given the weak tissue penetration of blue light and its potential phototoxicity to cells, Qiao et al. developed a NIR light-induced engineered bacterial expression system (NETMAP). Via genetic engineering of *Salmonella* strains and optogenetic regulation, the bacteria colonize tumors and proficiently produce ICIs nanobodies (PD-L1nb and CTLA-4nb) and the anticancer protein Azurin upon NIR light illumination, potently suppressing tumor growth in the absence of significant toxicity. In vivo investigations further revealed that the engineered bacteria-derived PD-L1nb and CTLA-4nb activated CD8^+^ T cells and NK cells, decreased the frequency of Treg cells, fostered M1 macrophage polarization, and augmented the antitumor immune response [120]. Biomineralization synthesis strategies further expand the dimensions of responsive design. Meng et al. used non-pathogenic sulfate-reducing bacteria (SRB) as a “bio-factory” for the in situ biomineralization of surface-decorated iron sulfide nanoparticles (FeS@SRB) (Figure 2D). This enabled active chemotaxis-driven targeted delivery to hypoxic tumors. Subsequent release of Fe^2+^ within the acidic TME instigated Fenton reactions generating hydroxyl radicals (•OH), which acted in concert with NIR photothermal effects to induce oxidative stress, ferroptosis, and apoptosis, resulting in significant suppression of diverse tumor growth. This biosynthetic approach boasts high delivery efficiency (an intravenous delivery efficiency of 50.5%, approximately 17-fold higher than chemically synthesized FeS@BSA), excellent biocompatibility, and in vivo safety, offering a translatable platform for non-pathogenic bacterium-mediated tumor-targeted therapy and the fabrication of green nanotherapeutics [9].

Thermal cues represent an ideal trigger for in vivo control of engineered bacteria owing to their superior tissue penetrability and versatile modes of generation (e.g., light, ultrasound, magnetic fields). Li et al. developed an *Escherichia coli* strain (HSB) controlled by the thermosensitive genetic circuit cI857, enabling precise thermal control for the treatment of breast cancer in vivo. In a murine subcutaneous 4T1 tumor model, chitosan-encapsulated HSB facilitated tumor-specific colonization, and thermal induction prompted the production of TNF-α, leading to marked suppression of tumor growth [121]. Fan and colleagues integrated synthetic biology with nanotechnology to synthesize gold nanoparticles on the surface of *Escherichia coli* MG1655 via biomineralization, endowing the bacteria with photothermal conversion capability, thereby achieving optothermal regulation of TNF-α expression under the control of a thermosensitive promoter. This system exhibited potent antitumor effects and a favorable safety profile in both in vitro and in vivo studies, surmounting the hurdles associated with targeted delivery of existing oral anticancer agents and offering the benefit of remote, non-invasive manipulation [27]. Additionally, the team also developed an engineered *Escherichia coli* strain (Ec-pE) that colonizes tumors and amplifies local H_2_O_2_ generation via overexpression of the NDH-2 enzyme. By combining with magnetic Fe_3_O_4_ nanoparticles, it catalyzes a Fenton-like reaction, efficiently converting H_2_O_2_ into highly toxic •OH, thereby inducing tumor cell apoptosis. This bioreactor enables a self-sustaining therapeutic Fenton-like reaction without the need for external H_2_O_2_ support, demonstrating significant potential for tumor therapy [122].

**Figure 3:**
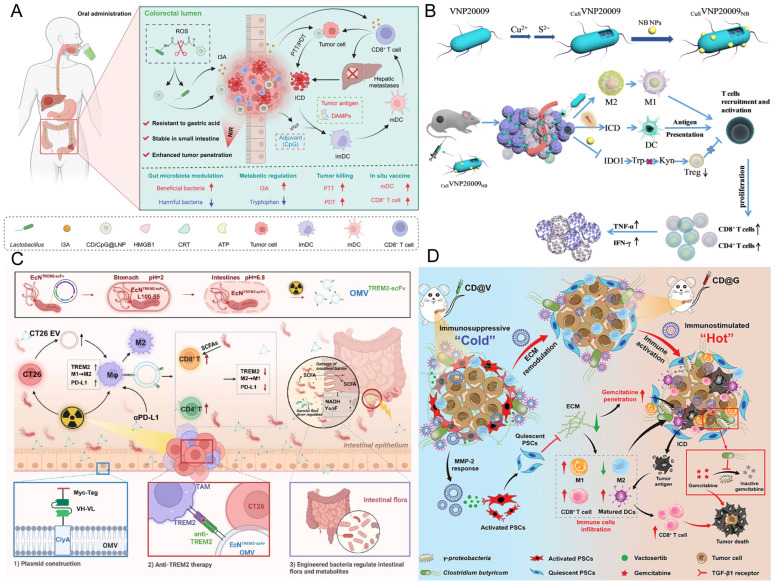
(**A**) Schematic illustration of orally administered CD/CpG@LNPs for optically controlled in situ vaccination and inhibition of tumor growth via modulation of microbial metabolism. Reproduced with permission from Ref. [123]. Copyright 2024, Wiley-VCH GmbH. (**B**) Schematic illustration of the construction of the bacteria-nano hybrid system CuS·VNP20009·NB and its mechanism in cancer therapy. Reproduced with permission from Ref. [112]. Copyright 2023, The Royal Society of Chemistry. (**C**) Schematic diagram of an ionization radiation-responsive delivery system. Reproduced with permission from Ref. [125]. Copyright 2025, Wiley-VCH GmbH. (**D**) Schematic illustration of the engineered bacterial-nano system CD@V remodeling the ECM to enhance chemotherapy in pancreatic cancer. Reproduced with permission from Ref. [127]. Copyright 2024, Wiley-VCH GmbH. ECM, extracellular matrix.

Beyond pH-responsive and photothermal-responsive systems, engineered bacteria strategies featuring metabolic responsiveness and radiation inducibility further augment the precision of targeted cancer therapy. These designs dynamically match drug release with pathological features, significantly enhancing efficacy and reducing off-target toxicity. The LR-S-CD/CpG@LNP platform developed by Xu et al. co-encapsulates photosensitive carbon dots (CD) and the immune adjuvant CpG via a thioether bond into mulberry leaf lipid nanoparticles, which are then anchored to the surface of *Limosilactobacillus reuteri* (Figure 3A). NIR illumination instigates a dual response: it simultaneously induces photothermal/photodynamic production of cytotoxic ROS and elicits ICD, while also liberating tumor neoantigens and CpG to constitute an in situ vaccine that fosters DCs maturation and CD8^+^ T cell recruitment. This strategy simultaneously suppressed primary foci and liver metastases in a rectal cancer model, with significantly reduced systemic toxicity, highlighting the crucial role of ROS response in local immune remodeling [123]. The pEcM-SE engineered bacteria designed by Cen et al. innovatively convert sialic acid metabolic signals into a therapeutic switch. This approach exploits the highly abundant sialoglycans on cancer cell surfaces as a chemical induction signal, allowing for selective bacterial production of the cytolysin hemolysin E (HlyE) within tumors, leading to tumor cell disruption. Simultaneously, sialidase enzymatic cleavage of sialoglycans on tumor cells effectively reverses immune suppression, facilitating the infiltration and activation of immune cells (T cells, NK cells, and M1 macrophages) into the tumor, markedly potentiating the antitumor response [124]. The bacteria-nano hybrid system CuS·VNP20009·NB achieves precise intervention in the TME through multi-level functional integration and pathological responsiveness design (Figure 3B). Using attenuated *Salmonella typhimurium* VNP20009 as a vector, this system decorates the bacterial surface with albumin nanoparticles (NB) capable of GSH-responsive release of the IDO-1 inhibitor NLG919. It enables targeted accumulation in hypoxic tumor niches, reprograms macrophages from an immunosuppressive M2 to a pro-inflammatory M1 state, and induces ICD through CuS-mediated photothermal effects upon NIR irradiation. This synergistically inhibits the IDO-1 pathway, enhances DCs maturation and CD8^+^/CD4^+^ T cell infiltration, potently suppresses tumor growth, and exhibits excellent biocompatibility [112]. The EcN modified by Wang et al. achieved precise therapy with dynamic regulation by radiation dose. Via genetic engineering, EcN was modified to be responsive to ionizing radiation and to produce a single-chain variable fragment (scFv) directed against TREM2 (Triggering Receptor Expressed on Myeloid Cells 2), thereby modulating macrophages, and synergizing with the ICIs αPD-L1 to enhance the radioimmunotherapy effect for low rectal cancer (Figure 3C). Simultaneously, this engineered bacterium can also be administered orally to modulate the gut microbiota, further optimizing the therapeutic outcome [125]. This multi-environmental response mechanism enables dynamic matching of drug release with tumor pathological features, reducing off-target toxicity by nearly 90% and improving efficacy by 3-fold in a pancreatic ductal adenocarcinoma model, providing a precise treatment paradigm for overcoming drug-resistant tumors [126].

However, it is imperative to acknowledge the inherent biological challenges that accompany the use of self-replicating entities. Chief among these are the concerns regarding long-term genetic stability of the engineered constructs during in vivo proliferation and the potential risk of horizontal gene transfer (HGT) to the host microbiota or environment. Plasmid systems for in vivo applications commonly face genetic instability, primarily manifested as cell growth inhibition due to metabolic burden, segregation errors caused by defects in replication initiation and partitioning mechanisms, and inactivation of therapeutic genes on high-copy plasmids due to point mutations, insertions, deletions, or recombination. Chromosomal integration into genomic safe sites using technologies such as Cre/lox, CRISPR-Cas homologous recombination, or CIChE, which eradicates plasmid segregation loss and significantly reduces the replication burden, is an effective strategy for enhancing genetic stability [128]. Furthermore, the introduction of toxin-antitoxin (TA) retention systems (e.g., hok/sok, ccdAB, vapBC) or tunable copy number plasmids can enhance plasmid maintenance through post-segregational killing or self-regulation of copy number [129]. Employing synthetic terminators and modular components to circumvent recombination triggered by repetitive elements further strengthens the assembly and sustained expression stability of multi-gene constructs [130]. Studies have indicated that synthetically engineered spatiotemporally controllable gene circuits (e.g., light-controlled or NIR-induced switching) can achieve precise gene expression regulation at tumor sites, balancing safety and efficacy enable precise regulation of gene expression within tumors, ensuring both safety and therapeutic effectiveness [131]. HGT poses a potential environmental hazard for the dissemination of engineered genetic elements into natural microbiota, since plasmids, transposons, or integrative cassettes can be transmitted between species through conjugation, transduction, or transformation [132]. To establish reliable biocontainment systems, research focus has shifted towards two major directions: synthetic auxotrophy dependent on non-standard amino acids and genome-wide codon reassignment. By deleting host genes essential for amino acid or phosphate uptake and introducing corresponding synthetic pathways, cells are engineered to survive only when exogenous non-standard amino acids or phosphates are supplied, creating a mandatory synthetic auxotrophy [133]; Further systematic compression and reassignment of unused codons, coupled with positive-feedback switches to achieve dual or multi-layer dependencies, builds a “genetic firewall” that makes functional HGT in natural environments nearly impossible [134].

### 3.3. Mitigating Toxicity and Enhancing Biocompatibility

Regarding the optimization of engineered bacteriotherapy for tumors, synthetic biology approaches have markedly improved therapeutic safety and controllability via multi-tiered strategies. Specifically, CRISPR-Cas systems enable targeted knockout of bacterial virulence factors, diminishing systemic inflammation induced by endotoxin release, whilst retaining their tumor-homing capability and antitumor efficacy. For example, Low et al. found that deleting the *msbB* gene in *Salmonella* resulted in lipid A losing its myristoylation modification, reducing TNF-α induction and virulence, while maintaining tumor-targeting ability and anti-tumor activity. This enhanced safety of the engineered strain represents a major advance and holds potential clinical value for the systemic administration of Gram-negative bacteria as anticancer agents [11]. Brockstedt et al. developed an attenuated live vaccine strain derived from *Listeria monocytogenes* (ΔactA/ΔinlB) through the deletion of two virulence determinants. This vaccine successfully decoupled immunogenicity from toxicity, maintaining immunogenic potency while reducing virulence by more than 1000-fold. Furthermore, it potently activated a strong and sustained antigen-specific T-cell immune response, overcame self-tolerance in murine tumor models, and significantly suppressed tumor growth while extending survival [135]. These genetic alterations constrained the bacterial replication and dissemination within healthy tissues, diminishing the risk of systemic infection by over 90%. Maciag et al. conducted the first Phase I clinical safety trial of attenuated live *Listeria monocytogenes* (Lm-LLO-E7) in patients with advanced cervical carcinoma. The results indicated that intravenous administration of this recombinant vaccine was overall manageable and safe, and elicited limited HPV-16 E7-specific T-cell responses in late-stage cervical cancer patients, although larger studies are required to confirm immunogenicity and therapeutic benefit [81]. A series of Phase I/II clinical trials have demonstrated that this *Listeria*-based immune platform technology consistently displays a manageable safety profile and preliminary antitumor immune activity across various solid tumors, including lung and prostate cancers [82,83,84,85]. Metabolism-dependent attenuation strategies further achieve biological containment through synthetic biology design. Thymidylate synthase gene (*thyA*) knockout strains proliferate only in the TME rich in free thymidine (concentration > 10 μM), while normal tissues cannot support their growth due to thymidine phosphorylase degradation [39]. The EcN strain SYNB1891, employing a dual auxotrophy strategy (thymine and diaminopropionic acid deficiency), showed good tolerability in a Phase I clinical trial, effectively preventing bacterial escape and uncontrolled growth in the host or environment [78]. Deletion of the *dapA* gene renders bacteria incapable of synthesizing diaminopimelic acid, precluding their survival in any tissue systemically. The dual safety switch (*thyA^−^/dapA^−^*) developed by Leventhal et al. achieved dual blockade of intra-tumoral/extra-tumoral proliferation; bacteria were cleared from normal tissues within 24 h, while intra-tumoral proliferation was unaffected [39]. This precise regulation requires balancing attenuation and efficacy. *Clostridium difficile* spore germination toxins retain direct killing of tumor cells, while the *C. difficile* auxotrophic design prevents its colonization in healthy tissues, demonstrating that attenuation and anti-tumor activity can be synergistically optimized [136]. Conversely, suicide gene systems (such as HSV-TK/GCV) induce programmed bacterial lysis through an external inducer, enabling precise eradication post-therapy and averting persistent infection [136]. To preclude genetic escape via HGT, genome recoding was implemented in *Escherichia coli* K12 by substituting serine codons (TCG/TCA) and incorporating an engineered tRNA system. Consequently, any acquired foreign genes would be rendered non-functional through mistranslation, thus obstructing the dissemination of toxic payloads (such as cytokine or toxin genes) to indigenous microbiota [137].

As mentioned earlier, the integration of controllable expression systems further enhances spatiotemporal specificity, enabling precise triggering and thereby reducing systemic exposure risk. For example, using TME-responsive promoters (e.g., hypoxia-inducible or acidic pH-responsive) to regulate the release of therapeutic payloads [70]. Surface modification strategies utilizing the photothermal effect of Au nanoparticles, by altering LPS structure or incorporating biocompatible coatings (e.g., polydopamine), reduce the immunogenicity of bacteria and extend their circulation half-life [138]. The magnetotactic bacterium *Magnetococcus* MC-1, after surface modification, exhibited enhanced tumor accumulation under the guidance of an external magnetic field, while reducing macrophage clearance and the release of inflammatory factors such as IL-6 and TNF-α [104]. Furthermore, by expressing tumor-specific ligands or antigens, engineered bacteria can reduce non-specific accumulation in organs such as the liver and spleen. Genetic engineering of EcN enabled specific adhesion to heparan sulfate proteoglycans (HSPGs) overexpressed on cancer cells, endowing the engineered bacteria with precise targeting ability and lessening effects on healthy tissues [139]. Moreover, optimizing the route of administration (such as intratumoral injection) directly confines bacterial spread, and integration with external technologies (like focused ultrasound) can further improve localized control [76]. For instance, a nanocomplex comprising *Bifidobacterium longum* and PFH nanoparticles administered via peritumoral injection enabled synergistic ablation through high-intensity focused ultrasound, thereby minimizing systemic adverse effects [140]. Recently, Zhang et al. reported a modular platform called spike engineering and retargeting (SPEAR), which utilizes an engineered bacterial nanoscale contraction injection system (*Photorhabdus* virulence cassette, PVC) for targeted delivery of diverse biomolecules in vitro and in vivo, ushering in a “modular era” for biological delivery [141]. The hierarchical integration of these strategies not only enhances the safety profile of engineered bacteria but also establishes a robust groundwork for clinical translation, although ongoing refinement in dosage control, genetic stability, and long-term surveillance is required to manage potential contradictions.

## 4. Synergistic Multimodal Therapy

Although engineered bacteria-nano systems show significant potential in tumor targeting and immune activation, the high heterogeneity of tumors at the molecular and tissue levels limits the efficacy of monotherapies. To address these constraints, combinatorial approaches that merge bacterial therapy with radiation, chemotherapy, immunotherapy, PDT, PTT, and other emerging modalities have emerged as a pivotal strategy to augment antitumor potency.

### 4.1. Combined with Chemotherapy

Although chemotherapy serves as a foundational oncologic intervention, its non-selective cytotoxic mechanism injures healthy cells, leading to substantial adverse effects including myelosuppression and gastrointestinal toxicity [142]. Moreover, drug penetration into solid tumors is inefficient, and nanovehicles dependent on the EPR effect frequently fail to access the deeply hypoxic core areas [143]. In combination regimens with chemotherapeutics, engineered bacteria can function as drug delivery systems enabling spatiotemporally controlled release of chemotherapeutic payloads. For instance, anaerobic Bifidobacterium species can be employed as smart drug vectors to deliver doxorubicin (DOX) nanoparticles to tumor hypoxic niches, markedly enhancing local drug levels whilst minimizing systemic exposure [144]. Yang et al. anchored DOX nanoparticles to the surface of engineered bacteria via electrostatic adsorption, achieving precise delivery to tumor colonization sites, while expressing HSulf-1 enzyme to degrade the tumor stromal barrier, forming an enzyme-chemotherapy synergistic therapy [145]. Xie et al. harnessed the self-propulsion of EcN to transport 5-fluorouracil (5-FU), functionalizing the bacteria with gold nanorods to enable photothermally regulated drug release, thereby improving spatiotemporal targeting accuracy [146]. Through genetic engineering, *Salmonella Typhimurium* was engineered for stable surface display of biotin molecules, enabling high-efficiency conjugation with streptavidin-decorated paclitaxel-loaded liposomes. This strategy markedly improved the tumor-targeted delivery efficiency of the chemotherapy drug and exhibited superior antitumor efficacy over single-agent therapy in both in vitro and in vivo studies [147,148]. Likewise, attenuated *Salmonella* can be coupled with temperature-sensitive liposomes carrying DOX using this platform. As shown in work by Ektate et al., externally applied local heating (e.g., via NIR irradiation) induces liposomal phase change and controlled drug release, enabling spatiotemporally regulated chemotherapy in models of colon cancer [149]. Furthermore, as a chemosensitizer, *Salmonella* A1-R can reactivate dormant cancer cells into the cell cycle, markedly increasing their susceptibility to cycle-specific agents like cisplatin and paclitaxel [150]. To overcome drug resistance by modulating the tumor microbiota, Shen et al. designed an oral symbiotic bacterium capable of delivering prodrugs to nasopharyngeal carcinoma sites, leveraging its robust colonization capacity to overcome physiological barriers [151]. Yao et al. developed a matrix metalloproteinase-2 (MMP-2) responsive engineered probiotic-nanomaterial system by utilizing *Clostridium butyricum* (CB) as a vehicle with surface-attached drug-loaded liposomes (Figure 3D). This system concurrently suppresses activated pancreatic stellate cells and remodels the dense ECM to improve tumor penetration of chemotherapeutics and immune cells, while the probiotic competitively inhibits γ-proteobacteria to diminish gemcitabine (GEM) metabolism and amplify GEM-triggered ICD, ultimately significantly improving the chemo-immunotherapy effect for pancreatic cancer [127]. Zheng’s team utilized bacteriophages to specifically eradicate resistance-promoting *Fusobacterium nucleatum*, concomitantly delivering chemotherapy-loaded nanoparticles to counteract the immunosuppressive milieu, markedly improving the efficacy of first-line chemotherapy in colorectal cancer [152]. This combined strategy leverages the microenvironment-responsive properties of bacteria to accomplish precise and regulated drug release, enhancing intratumoral drug bioavailability while minimizing systemic toxic exposure. It effectively surmounts the limitations of conventional chemotherapy resistance and enables profound targeted delivery that surpasses the constraints of the EPR effect.

### 4.2. Combined with Radiotherapy

Radiotherapy works by damaging the DNA structure of tumor cells with high-energy radiation, thereby inhibiting their proliferative capacity to achieve therapeutic goals. However, similar to chemotherapy, radiotherapy can inflict collateral damage on adjacent healthy tissues during tumor cell eradication, leading to significant adverse effects [153]. Furthermore, the diminished radiosensitivity inherent to the hypoxic TME, coupled with its immunosuppressive nature, predisposes tumors to develop radiation resistance [154]. Engineered bacteria, leveraging their natural tumor-targeting ability and hypoxia tropism, can precisely colonize the tumor core region, enhancing the targeting and sensitivity of radiotherapy. Firstly, as radiosensitizers and imaging-guided carriers, engineered bacteria can be loaded with functional molecules to improve the TME and enable real-time monitoring. For example, *Salmonella* carrying aggregation-induced emission luminogens can specifically accumulate in tumor tissue, efficiently producing ROS under X-ray irradiation, significantly enhancing radiotherapy killing efficiency, while guiding precise radiotherapy targeting via fluorescence imaging [155]. Secondly, engineered bacteria can serve as delivery vectors for radionuclides, achieving long-term, high-dose internal irradiation within the tumor. Attenuated *Listeria monocytogenes* conjugated with Rhenium-188 successfully targeted metastatic lesions, substantially minimizing radiotoxicity to normal tissues [30]. Inactivated bacterial vectors tagged with ^125^I or ^131^I not only achieve prolonged intratumoral retention for localized emission of ionizing radiation but also potentiate innate and adaptive immunity, eliciting an “in situ vaccine” effect [156]. Moreover, engineered bacteria mediate indirect radiosensitization through the secretion of factors that remodel the TME. For instance, engineered Salmonella strains designed for intratumoral delivery of nattokinase can degrade the tumor ECM, enhance perfusion, and ameliorate hypoxia, consequently overcoming radioresistance [157]. Research by Zhang et al. further confirmed that this strategy can effectively reduce tumor stiffness and enhance the killing depth of radiotherapy against deep-seated tumor cells [158]. It is noteworthy that the synergistic advantage of combination therapy is manifested not only in improving the precision and efficacy of radiotherapy but also in activating systemic anti-tumor immunity. The inflammatory response triggered by bacterial colonization can recruit immune cells such as macrophages and neutrophils to infiltrate, and promote M1 macrophage polarization via the TLR4/MyD88 signaling pathway, reversing the immunosuppressive TME [34]. Simultaneously, radiotherapy-induced release of tumor antigens and bacteria-mediated immune stimulation form an “ICD-antigen presentation” closed loop, enhancing the abscopal anti-tumor effect [159]. However, clinical translation still requires balancing efficacy and safety. For instance, while Salmonella VNP20009 has confirmed tumor-targeting capabilities, administration at higher doses can still induce toxicities like thrombocytopenia, underscoring the need for further genetic engineering to optimize strain attenuation and controlled-release strategies. In conclusion, the integration of engineered bacteria with radiotherapy offers a novel paradigm to overcome the limitations of solid tumor therapy via a tripartite synergy encompassing radiosensitization, drug carriage, and immune modulation.

### 4.3. Combined with Immunotherapy

Although immunotherapy has achieved breakthroughs in cancer treatment, it still faces clinical bottlenecks such as limited response rates and an immunosuppressive TME. The introduction of engineered bacteria provides an innovative solution to overcome these obstacles [77]. Firstly, as immune activators and antigen delivery vectors, they can efficiently activate both innate and adaptive immunity. For example, attenuated double-deleted *Listeria* expressing tumor antigens (LADD-Ag) can promote CD8^+^ T cell infiltration and reduce Treg levels, transforming the immunosuppressive TME into an inflammatory TME, thereby inducing tumor regression [160]. Wang et al. reported a novel strategy using injected, modified flagellated bacteria (VNP20009) to actively transport tumor antigens to tumor margin tissues, thereby activating functionally competent marginal DCs and initiating a systemic anti-tumor immune response. In a 4T1 breast cancer metastasis model, flagellin served as a natural immune adjuvant, synergistically enhancing the immunogenicity of tumor antigens and the activation level of DCs, significantly prolonging mouse survival and reducing lung metastases [161]. Zhang et al. engineered an attenuated *Salmonella Typhimuriumstrain* (SAM) programmed to secrete a fusion protein combining IL-15 and Vibrio *vulnificus* FlaB in response to specific cues. This increased the M1 macrophage population, providing dual immune activation, which effectively suppressed tumor progression and enhanced animal survival [162]. *Engineered Escherichia coli* delivering STING agonists (such as cyclic di-glycerate) can target APCs, enhancing type INF-I production and antigen presentation efficiency. Secondly, engineered bacteria can serve as in situ production factories for ICIs, overcoming the toxicity of systemic administration. Non-pathogenic *Escherichia coli* strain uses a SLC to specifically release a CD47 nanobody antagonist within the TME, significantly enhancing macrophage phagocytic function and inducing durable systemic immunity, with lower toxicity than traditional intratumoral or intravenous injection [28]. Similarly, attenuated *Escherichia coli* stably releases anti-PD-L1 and anti-CTLA-4 nanobodies, demonstrating high tumor suppression efficacy in weakly immunogenic models [47]. Additionally, nanoliposome-bacteria hybrid systems utilize bacterial chemotaxis to achieve macrophage infiltration and polarization, and express CD47 antibodies in the hypoxic TME to enhance activity [108]. Meanwhile, bacterial outer membrane vesicles (OMVs), as natural carriers, can be engineered to deliver immunomodulators (e.g., PD-1 antagonists), activating anti-tumor immunity [163]. Furthermore, synthetic biology-driven multifunctional synergistic designs further expand the boundaries of efficacy. For example, engineering bacteria to produce melanin (a photothermal agent) and surface-anchor PD-1 antibodies can achieve photothermally triggered high-intensity anti-tumor immunity [164]. Regarding metabolic modulation, engineered EcN convert ammonia within the TME into L-arginine, markedly amplifying T cell-mediated antitumor responses and acting synergistically with PD-L1 blockade therapy [46]. Yuan et al. developed a programmed immunobiotic platform (EcN@Nbs-NP@API-1) by utilizing EcN as a vehicle coated with pH-sensitive nanoparticles encapsulating the Pin1 inhibitor API-1. This platform is designed to inhibit Pin1, reprogram the fibrotic and immunosuppressive TME, and locally produce anti-PD-L1 nanobodies [113]. Simultaneously, bacterial vaccines can effectively promote DCs maturation and macrophage polarization, strengthening the immune response [165]. Chang et al. constructed a tumor-specific targeting *Salmonella Typhimurium* strain DB1, which exploits IL-10 secreted by tumor-associated macrophages to create a high IL-10 receptor expression state. This balances immune evasion mechanisms and potentiates antitumor immunity, resulting in significant inhibition of tumor growth and metastasis [166].

### 4.4. Combined with Photodynamic Therapy and Photothermal Therapy

PDT, as a non-invasive cancer treatment strategy, relies on PS generating singlet oxygen (^1^O_2_) under light of specific wavelengths to destroy tumor cells [167]. However, the clinical translation of PDT faces challenges such as insufficient targeting of PS and limited light penetration depth. Engineered bacteria serve as novel vectors that, through synergistic interaction with PDT, markedly improve tumor homing, therapeutic penetration, and immune potentiation. Engineered bacteria can serve as efficient carriers for PS or in situ production factories. For instance, Wu et al. loaded PS-coated nanoparticles onto the surface of *Escherichia coli* via electrostatic adsorption. Under light illumination, they not only produced ROS to directly kill cancer cells but also disrupted the bacterial membrane with ROS to release intracellular proteins, unveiling a novel strategy for protein-based drug delivery [168]. Engineered bacteria can be designed as in situ PS biosynthesis factories. As demonstrated by Chen’s team, genetic engineering of *Escherichia coli* to incorporate a 5-ALA (δ-aminolevulinic acid) biosynthetic pathway allowed for sustained intratumoral generation of the endogenous PS protoporphyrin IX, enabling spatiotemporally controlled PDT upon light irradiation [169]. Furthermore, engineered bacteria can actively ameliorate TME hypoxia, mitigating the suppression of ^1^O_2_ generation. Engineered Escherichia coli expressing human catalase can catalyze the conversion of endogenous H_2_O_2_ to O_2_, which upon NIR light excitation generates ^1^O_2_, directly enhancing PDT efficacy [114]. Similarly, photosynthetic bacteria (PSB) (e.g., cyanobacteria) continuously supply oxygen through photosynthesis; when combined with black phosphorus nanosheets (BPNSs) to form a Cyan@BPNSs system, they achieve a cascade reaction of in situ O_2_ supply and ^1^O_2_ generation [170]. It is noteworthy that engineered bacteria can be transformed into endogenous biological light sources to overcome penetration depth limitations. Yang et al. introduced a luciferase expression plasmid into attenuated Salmonella. Upon intratumoral administration and fixation of the luciferase substrate, a homogeneous bioluminescent source was established, enabling persistent activation of the PS Ce6 and resulting in inhibition of deep-seated tumors [171].

The clinical application of PTT has long been limited by bottlenecks such as poor water solubility of traditional photothermal agents, rapid systemic clearance, and significant toxic side effects. Additionally, nanodelivery systems struggle to achieve effective enrichment at tumor sites due to complex biological barriers, urgently requiring innovative strategies to break through the delivery and efficacy dilemma. Engineered bacteria, leveraging their inherent tumor tropism and microenvironment adaptability, provide a multi-dimensional synergistic solution for PTT. Intravenously administered attenuated *Salmonella* strains exhibit specific colonization of tumors. They provoke local inflammation that compromises vascular integrity, leading to enhanced tumor pigmentation which improves NIR light absorption, culminating in effective laser ablation [172]. Sun et al. constructed an engineered bacterial biohybrid (eVNP@AuNFs) using attenuated *Salmonella* VNP20009 as a platform, with flower-like gold nanoparticles (AuNFs) adsorbed on its surface and carrying shRNA plasmids targeting CD47 and HSP90. The results showed that downregulating HSP90 reduces cellular heat tolerance, enhancing the lethal effect of PTT and promoting apoptosis. This system nearly completely suppressed tumor growth in a 4T1 murine model [115]. PSB utilize their natural chlorophyll components to form strong NIR absorption characteristics. Their facultative anaerobic nature allows for precise targeting of hypoxic tumor areas, forming an autonomous photothermal conversion platform [173]. Liu et al. covalently linked the PSB *Synechococcus* 7942 (Syne) to ICG-loaded human serum albumin nanoparticles (HSA/ICG) through amide bonds, creating an S/HSA/ICG biomimetic system. The photosynthetic activity of Syne enabled sustained in situ oxygen generation within tumors to ameliorate hypoxia, markedly potentiating the tumoricidal efficacy of PTT in a 4T1 metastatic model [116]. Regarding carrier function, *Salmonella* strains were covalently conjugated to biocompatible photothermal agents through amide linkages, while *Bifidobacterium* species were loaded with Ag_2_S quantum dots via electrostatic adsorption. These carrier strategies substantially increased the tissue-penetration depth of the photothermal agents, ultimately eradicating bulky solid tumors [174,175]. Even more innovative is the strategy of bacteria-mediated in situ generation of photothermal agents. *Escherichia colis* electively reduces a supramolecular complex based on a perylene diimide derivative (CPPDI), leading to the in situ formation of the highly efficient photothermal agent CPPDI radical anion (CRAs) within the tumor, enabling precisely controlled PTT with exceptional spatiotemporal accuracy [176]. Through synergistic optimization of genetic engineering and surface modification, such as the ternary photosensitizer-bacteria composite system constructed by Guo et al., it can simultaneously activate photoacoustic imaging guidance and PTT under single-wavelength (808 nm) light illumination, significantly improving tumor regression rates and prolonging survival [177]. Such multifunctional integrated designs can also effectively avoid the operational complexities such as wavelength mismatch in traditional combined phototherapy. Hyperbaric oxygen pretreatment can enhance bacterial penetration and colonization within tumors, improving the subsequent efficacy of PTT. Xu et al. found that non-invasive hyperbaric oxygen (HBO) can effectively deplete the dense ECM of tumors, thereby significantly increasing the penetration and accumulation of engineered EcN modified with the photothermal dye cypate (EcN-cypate) within tumors, and promoting an anti-tumor immune response through PTT-induced ICD [178]. Through multimodal synergistic therapeutic mechanisms, engineered bacteria not only overcome the pharmacokinetic defects of traditional photothermal agents but also achieve efficient ablation of deep tumors and precise imaging monitoring, providing a breakthrough paradigm for the clinical translation of PTT.

### 4.5. Other Synergistic Strategies

Based on current research progress in engineered bacterial cancer therapy, other combination treatment strategies are demonstrating significant potential through multidisciplinary innovation. Magnetic navigation technology manipulates engineered bacteria loaded with magnetic nanoparticles (e.g., *Magnetococcus* MC-1) via an external magnetic field, achieving precise tumor-targeted positioning and dynamic distribution control, effectively overcoming intratumoral heterogeneity barriers and enhancing treatment penetration [179]. In combination with cell therapy, engineered probiotics can guide CAR-T cells to target solid tumors, for example, by synthesizing CAR targets and bacterial fusion proteins (e.g., HBD) for local release at the tumor site, enhancing T cell infiltration and inducing antigen-independent cell death while stimulating systemic anti-tumor immunity to suppress distal metastasis [180]. Sun et al. developed an innovative drug delivery platform called engineered bacteria-outer membrane vesicle complexes, which utilize bacterial-specific ABC transporters to carry therapeutic agents across the blood–brain barrier, targeting and deeply penetrating tumor tissue. Under 808 nm laser irradiation, the nanoparticles generate active heat to destroy both tumor and bacterial cells, promoting the release of tumor-associated antigens and PAMPs, activating innate and adaptive immune responses. Compared to bare nanoparticles or bacterial therapy alone, this system significantly inhibited tumor growth, prolonged mouse survival, and effectively cleared residual bacteria from the body after treatment, demonstrating good safety and therapeutic potential [181]. Liu et al. proposed modifying bacteria with biomimetic coating technology to synergistically activate anti-tumor and anti-viral immunity, achieving dual therapeutic and preventive efficacy [182]. Furthermore, the engineered bacterium DB1 designed by Rong et al. induces tertiary lymphoid structure (TLS) maturation via the IL-10 signaling axis, promotes CD8^+^ T cell activation and infiltration, and significantly inhibits tumor growth in colon cancer and melanoma models. This strain simultaneously remodels the immunosuppressive TME, increases cytotoxic protein secretion, and exhibits a synergistic effect with PD-L1 inhibitors [183]. These innovative strategies, through spatiotemporal precise manipulation and cascade biological reactions, provide a new paradigm for overcoming the limitations of traditional therapies, but further optimization of bacterial safety, genetic stability, and clinical translation pathways remains necessary.

## 5. Future Directions and Translational Prospects

Based on current research progress, engineered bacteria-nano hybrid system cancer therapy has evolved from empirical application to a precision regulation strategy integrating synthetic biology, material science, and immunology. However, Bacillus *Calmette-Guérin* (BCG) remains the only bacteria-based therapy approved by the Food and Drug Administration (FDA), used as first-line treatment for non-muscle-invasive bladder cancer [86,87,184]. However, several engineered strains (e.g., attenuated *Salmonella* VNP20009, *Listeria* SYNBY1891) have entered clinical trials, confirming the safety of intravenous or intratumoral administration and preliminary tumor colonization effects [79,80,88,89]. Yet, the translational development of engineered bacteriotherapy for cancer continues to encounter several safety-related hurdles. The innate toxicity and genetic instability of the bacterial vectors represent critical challenges. The inherent pathogenic potential of the bacteria can lead to serious adverse events, as observed in clinical studies where high doses of *Salmonella* VNP20009 provoked thrombocytopenia, persistent bacteremia, and multi-organ toxicity [90]. Simultaneously, recombinant plasmids carried by engineered bacteria pose a mutation risk, potentially leading to loss of tumor-penetrating ability before exerting therapeutic effects. Moreover, drug resistance issues pose a dual threat. Many therapeutic strains already possess multidrug resistance, not only directly increasing the risk of treatment failure, secondary infections, and even patient death but also potentially leading to horizontal transfer of resistance genes to other species through environmental escape, disrupting ecological balance. Potential infection risks are also a significant concern, as live bacterial products might colonize implanted medical devices, establishing infectious niches that are challenging to clear.

The future advancement of engineered bacteria-nano hybrid systems for cancer therapy necessitates a deeper understanding of bacterium-host interactions and the development of real-time monitoring systems (e.g., biosensors) to enable safe, closed-loop management across the entire therapeutic course. In the field of precision therapy, combining AI-driven tumor neoantigen prediction with CRISPR technology to screen high-affinity carriers (e.g., *Bifidobacterium* delivering KRAS mutant antigens) will promote breakthroughs in personalized vaccines, activating specific anti-tumor immune responses through customized antigen delivery to overcome tumor heterogeneity limitations. Simultaneously, optimization of closed-loop control systems is crucial for balancing efficacy and safety. By designing lactate-responsive sensors to dynamically regulate IL-12 expression and inhibit TNF-α toxicity, combined with dual-feedback gene circuits to achieve adaptive regulation of drug release, real-time adaptation of therapeutic effects and enhanced safety can be achieved. Additionally, developing multi-responsive intelligent regulation systems (pH/temperature/biomarker-responsive promoters) will enhance the spatiotemporal controllability of bacterial behavior and reduce off-target effects. Interdisciplinary integration will accelerate technological innovation. On one hand, integrating imaging technologies such as ultrasound and MRI can enable remote visual manipulation of bacterial localization, colonization, and drug release, improving tumor targeting accuracy. On the other hand, novel carriers such as bacteria-nanorobots and bacteria-oncolytic virus complexes can overcome physiological barriers like the blood–brain barrier. Their combination with immunotherapy (e.g., CAR-T, ICIs) or radiotherapy can remodel the immunosuppressive TME, achieving synergistic effects through local immune activation enabled by 3D-bioprinted bacterial scaffolds. Microbial-AI consortia focus on diagnostic innovation. Engineered bacteria equipped with genetic circuit sensors can detect tumor DNA mutations in real time, promoting non-invasive early diagnosis and guiding dynamic treatment adjustments. Looking forward, the clinical translation of this promising technology hinges not only on technical optimization but also on its potential integration into mainstream oncological guidelines and healthcare economics.

## Figures and Tables

**Table 1:** Clinical studies on the engineered bacterial-nano hybrid system for cancer therapy.

Bacteria	Code	Cancer Type	Number	Phase	Treatment	Outcome	Limitations	Ref.
*Clostridium novyi*	novyi-NT	Solid tumor	24	I	Single IT injection, dose escalation (10^4^–3 × 10^6^ spores)	MTD: 1 × 10^6^ spores. Tumor lysis in 42% (10/24). DLTs in patients with large tumors (>8 cm).	DLTs: Grade 4 sepsis (*n* = 2), gas gangrene (*n* = 1).	[76]
*Clostridium novyi*	novyi-NT	Breast cancer; melanoma	16	Ib	IT + IV Pembrolizumab	Confirmed ORR: 25% (4/16). DCR: 69%. MTD: 1 × 10^6^ spores.	Grade 3 DLT (abscess); AEs: fever (19%), leukopenia (13%).	[77]
*Escherichia coli*	SYNB1891	Metastatic solid neoplasm	32	I	IT, mono or combo with atezolizumab	Well-tolerated. STING pathway activation confirmed. No bacteremia.	CRS (*n* = 5), one of which met the criteria for DLT.	[78]
*Listeria*	ANZ-100	Metastatic liver cancer	9	I	IV, single dose escalation	MTD: 1 × 10^9^ CFU. DLT: CRS at 1 × 10^10^ CFU.	Transient flu-like symptoms; limited immune persistence.	[79]
*Listeria*	CRS-207	Solid tumors expressing mesothelin	17	I	IV, multiple doses	37% of subjects survived ≥15 months.	Transient lymphopenia, hypophosphatemia, fever; CRS risk.	[79]
*Listeria*	CRS-207	Metastatic pancreatic cancer	93	II	IV infusion (1 × 10^9^ CFU)	mOS > 3.9 months. Manageable toxicity (fever, lymphopenia).	AEs included transient fever, lymphopenia, elevated liver enzymes.	[80]
*Listeria*	Lm-LLO-E7	Cervical cancer	15	I	IV, second dose after 3 weeks	DLT (hypotension) at highest dose (10 × 10^9^ CFU). Lower doses well-tolerated.	DLT in 3 patients (hypotension); transient flu-like symptoms.	[81]
*Listeria*	JNJ-809	Metastatic castration-resistant prostate cancer	26	I	IV, two dose groups	RP2D: 1 × 10^9^ CFU. Limited antigen-specific T-cell response noted.	Grade 1–2 AEs: chills (92%), fever (81%), fatigue (62%).	[82]
*Listeria*	ADXS11-001	Advanced cervical cancer	109	I	IV, mono or combo with Cisplatin	12-month OS rate: 34.9%. 18-month OS rate: 24.8%. Potential long-tail benefit.	Most frequent drug-related AEs: chills and fever.	[83]
*Listeria*	ADXS11-001	Squamous cell carcinoma of the anorectal canal	36	II	IV, q3w	mOS: 12.6 month. mPFS: 2.0 months. Grade 3 AEs: 27.8%.	Grade 3 CRS (*n* = 3); grade 4 respiratory failure (*n* = 1).	[84]
*Listeria*	JNJ-757	Non–small cell lung cancer	30	I	IV, mono or combo with Nivolumab	RP2D: 1 × 10^9^ CFU.	Pneumonitis risk (2 fatal cases); low T cell response.	[85]
*Mycobacterium bovis*	VPM1002BC	Bladder cancer	40	I/II	Intravesical	1-year RFS: 49.3%.	Genitourinary infections (33.3%); risk of undetected metastases.	[86]
*Mycobacterium bovis*	VPM1002BC	Bladder cancer	6	I	Intravesical	Well-tolerated. No DLTs. Most AEs were Grade 1–2 UTI or asymptomatic bacteriuria.	Asymptomatic UTI (*n* = 4); prostatitis (*n* = 1).	[87]
*Salmonella typhimurium*	VNP20009	Head and neck cancer; oesophageal cancer	3	I	TAPET-CD (CEA-directed) infusion	Persistent tumor colonization (≥15 days) & 5-FC to 5-FU conversion in 2 patients.	No MTD reached; no objective tumor regression.	[88]
*Salmonella typhimurium*	VNP20009	Metastatic melanoma	4	I	IV infusion (4-hr)	Well-tolerated; one patient disease-free at 3 months.	Transient hypoxia, hypophosphatemia, hyperuricemia.	[89]
*Salmonella typhimurium*	VNP20009	Metastatic melanoma; metastatic renal cell carcinoma	24	I	IV bolus (30-min), escalation	MTD: 3 × 10^8^ CFU/m^2^. DLTs at 1 × 10^9^ CFU/m^2^ (CRS).	Dose-dependent thrombocytopenia (≥1 × 10^9^ CFU/m^2^); persistent bacteremia.	[90]
*Salmonella typhimurium*	VXM01	Advanced pancreatic cancer	27	I/II	Oral, two dose groups	Enhanced VEGFR2-specific T-cell response (≥3 × in 8/16 patients), stronger in high-dose group.	Lymphocyte decrease (27.8%), neutrophil increase (16.7%), diarrhea (22.2%).	[74]
*Salmonella typhimurium*	Saltikva	Metastatic gastrointestinal cancers	22	I	Oral, single dose escalation	MTD: 1 × 10^10^ CFU. No DLTs or drug-related SAEs. No bacteremia.	No evidence of partial or complete response.	[75]

AE, adverse event; CFU, colony-forming unit; CRS, cytokine release syndrome; DCR, disease control rate; DLT, dose-limiting toxicity; IT, intratumoral; IV, intravenous; mOS, median overall survival; mPFS, median progression-free survival; MTD, maximum tolerated dose; ORR, objective response rate; RFS, recurrence-free survival; RP2D, recommended phase II dose; SAE, serious adverse event; UTI, urinary tract infection.

**Table 2:** Applications and mechanisms of the engineered bacteria-nanomaterial hybrid system in cancer therapy.

Hybrid System	Engineered Bacteria	Nanomaterial	Loaded Agent	Cancer Type	Key Mechanisms	Key Outcomes	Primary Advantage	Ref.
FeS@SRB	Sulfate-reducing bacteria (SRB)	FeS nanoparticles	None (self-releases Fe^2+^)	Breast cancer and melanoma mouse models	FeS releases Fe^2+^ in acidic TME, generates ROS (●OH) via Fenton reaction	Significant tumor growth inhibition and extended survival; high tumor accumulation rate (approx. 50.5%)	Serves as both biosynthetic factory and active carrier	[9]
HRB@LM	Attenuated *Escherichia coli* MG1655	LM	M-CSF	Colon, lung, pancreatic cancer mouse orthotopic and metastasis models	LM releases M-CSF in reductive TME, recruits and promotes macrophage polarization towards M1 type	Inhibition of primary and distant tumors and metastasis; increased intratumoral M1 macrophages and CD8^+^ T cells	In situ continuous production of therapeutic protein, reducing systemic exposure and toxicity	[108]
DL@SFEc+	Engineered *Escherichia coli* with high CySS uptake and decomposition capability	DMXAA-loaded liposome	DMXAA (vascular disrupting agent)	Spontaneous intestinal adenocarcinoma and pancreatic cancer mouse models	DMXAA disrupts tumor neovasculature, blocks nutrient supply; engineered bacteria consume CySS, disrupting intracellular redox homeostasis, inducing lipid peroxidation and ferroptosis	Near-total tumor regression; no significant weight loss or major organ toxicity	Synergistic strategy of vascular disruption and metabolic exhaustion, enhancing antitumor efficacy	[109]
VASAM@BTO	Veillonella atypica (VA)	SAM-coated BaTiO_3_ piezoelectric nanocubes (BTO)	BTO (piezoelectric material)	Colorectal cancer mouse model	Ultrasound activates BTO to generate ROS (O_2_^−^, OH), produce CO, deplete GSH, and oxidize lactate	Significant tumor growth inhibition (up to approx. 90%) and extended survival	Piezocatalytic and microbial metabolic synergy depletes lactate and produces multiple cytotoxic stress products (ROS/CO) for direct tumor killing and immune activation	[110]
PP3244@FeZT	Low-pathogenicity *Escherichia coli* DH5α	Fe-doped metal-organic framework nanoparticles (Fe-ZT)	Tirapazamine (TPZ, hypoxia-activated chemotherapeutic agent)	Breast cancer mouse model	Fe-ZT exhibits peroxidase-like activity, converting H_2_O_2_ to ●OH; TPZ is reductively activated under hypoxia/acidosis to produce cytotoxic products	Significant tumor growth inhibition, with tumors nearly disappearing by day 6	Couples genetic engineering and materials engineering to form an amplified therapeutic chain within the TME	[111]
CuSVNP20009NB	Attenuated Salmonella typhimurium VNP20009	Albumin nanoparticles (NB NPs)	NLG919 (IDO-1 inhibitor)	Melanoma mouse model	NB NPs release NLG919 in high GSH TME, inhibiting IDO-1 pathway, alleviating immune suppression	Increased intratumoral CD4^+^/CD8^+^ T cell infiltration; elevated IFN-γ, TNF-α levels	Multimodal synergistic regulation of TME (targeting, macrophage reprogramming, ICD induction, IDO inhibition)	[112]
EcN@NbsNP@API-1	*Escherichia coli* Nissle 1917	pH-sensitive dextran-based nanoparticle	API-1 (Pin1 inhibitor)	Pancreatic cancer mouse model	Pin1 inhibition reduces CAF and collagen deposition, upregulates PD-L1 expression, promotes CD8^+^ T cell infiltration	Significantly reduced tumor burden, extended median survival from 32 days to 48 days	Remodels immunosuppressive TME and performs immune checkpoint blockade	[113]
E. Coli (p)/pDA/Ce6	*Escherichia coli* DH5α (non-pathogenic)	Polydopamine-coated Ce6 (pDA/Ce6)	Catalase gene plasmid + photosensitizer Ce6	Mouse osteosarcoma model	Catalase expression generates O_2_, enhancing PDT; simultaneous PTT (pDA) and PDT (Ce6) synergistically kill tumor cells	Tumor inhibition rate > 95%; no significant histopathological or biochemical toxicity observed	Bacterial targeting and enrichment with endogenous oxygen generation, significantly enhancing PDT/PTT synergistic efficacy	[114]
eVNP@AuNFs	Attenuated Salmonella typhimurium VNP20009	Polydopamine-coated gold nanoflowers (AuNFs)	CD47 shRNA plasmid + HSP90 shRNA plasmid (genetic drugs)	Breast cancer mouse	NIR-II PTT induces ICD; CD47 and HSP90 gene silencing enhances phagocytosis and antitumor immunity	Near-complete tumor growth inhibition, induced necrosis/apoptosis, reduced recurrence, and generated immune memory	Bacterially delivered genetic drugs and photothermal materials synergistically enhance innate and adaptive immunity	[115]
S/HSA/ICG	Photosynthetic cyanobacterium Synechococcus elongatus	HAS nanoparticles loaded with indocyanine green (ICG)	ICG (photosensitizer)	Triple-negative breast cancer mouse	Bacterial photosynthesis produces O_2_, alleviating tumor hypoxia; O_2_ enhances ICG-mediated PDT, inducing strong ICD and activating antitumor immunity	Significant inhibition of primary tumor growth and reduced lung metastasis; enhanced tumor-infiltrating T cells	Photosynthetic continuous O_2_ supply enhances PDT, effectively overcoming the limitations of hypoxic TME	[116]

CAF, cancer-associated fibroblast; CySS, cystine; GSH, glutathione; HSA, human serum albumin; ICD, immunogenic cell death; IDO-1, indoleamine 2,3-dioxygenase 1; LM, liposome; NIR-II, near-infrared region II; PD-L1, programmed death-ligand 1; PDT, photodynamic therapy; PTT, photothermal therapy; ROS, reactive oxygen species; SAM, Staphylococcus aureusmembrane; TME, tumor microenvironment; TNF-α, tumor necrosis factor-alpha.

## Data Availability

No new data were created or analyzed in this study.

## Abbreviations

The following abbreviations are used in this manuscript:

AHL	Acyl-homoserine lactone
APCs	Antigen-presenting cells
CAR-T	Chimeric antigen receptor T cell
ClyA	Cytolysin A
DCs	Dendritic cells
DOX	Doxorubicin
EcN	*Escherichia coli* Nissle 1917
EPR	Enhanced permeability and retention
FlaB	Flagellin B
GEM	Gemcitabine
HGT	Horizontal gene transfer
ICIs	Immune checkpoint inhibitors
ICD	Immunogenic cell death
LPS	Lipopolysaccharide
MDSCs	Myeloid-derived suppressor cells
NIR	Near-infrared
PAMPs	Pathogen-associated molecular patterns
PDT	Photodynamic therapy
PTT	Photothermal therapy
PS	Photosensitizers
QS	Quorum sensing
ROS	Reactive oxygen species
SLC	Synchronized lysis circuit
TME	Tumor microenvironment
TLRs	Toll-like receptors
Tregs	Regulatory T cells
